# Real-Time Object Detection for Autonomous Solar Farm Inspection via UAVs

**DOI:** 10.3390/s24030777

**Published:** 2024-01-25

**Authors:** Javier Rodriguez-Vazquez, Inés Prieto-Centeno, Miguel Fernandez-Cortizas, David Perez-Saura, Martin Molina, Pascual Campoy

**Affiliations:** 1 Computer Vision and Aerial Robotics Group, Universidad Politécnica de Madrid (CVAR-UPM), 28040 Madrid, Spain; ines.prieto@upm.es (I.P.-C.); miguel.fernandez.cortizas@upm.es (M.F.-C.); david.perez.saura@upm.es (D.P.-S.); martin.molina@upm.es (M.M.); pascual.campoy@upm.es (P.C.); 2 Centre for Automation and Robotics C.A.R. (UPM-CSIC), Calle Jose Gutierrez Abascal 2, 28006 Madrid, Spain; 3 Department of Artificial Intelligence, Universidad Politécnica de Madrid (UPM), 28031 Madrid, Spain

**Keywords:** keypoint detection, onboard processing, embedded platforms, active learning, autonomous navigation, neural networks, uncertainty estimation

## Abstract

Robotic missions for solar farm inspection demand agile and precise object detection strategies. This paper introduces an innovative keypoint-based object detection framework specifically designed for real-time solar farm inspections with UAVs. Moving away from conventional bounding box or segmentation methods, our technique focuses on detecting the vertices of solar panels, which provides a richer granularity than traditional approaches. Drawing inspiration from CenterNet, our architecture is optimized for embedded platforms like the NVIDIA AGX Jetson Orin, achieving close to 60 FPS at a resolution of 1024 ×1376 pixels, thus outperforming the camera’s operational frequency. Such a real-time capability is essential for efficient robotic operations in time-critical industrial asset inspection environments. The design of our model emphasizes reduced computational demand, positioning it as a practical solution for real-world deployment. Additionally, the integration of active learning strategies promises a considerable reduction in annotation efforts and strengthens the model’s operational feasibility. In summary, our research emphasizes the advantages of keypoint-based object detection, offering a practical and effective approach for real-time solar farm inspections with UAVs.

## 1. Introduction

Unmanned aerial vehicles (UAVs) have emerged as a viable solution for monitoring and inspecting solar farms, owing to their ability to cover large areas in relatively short periods [1,2,3].

While industrial inspections traditionally rely on semi-automated procedures, where pilots direct drones over predefined areas, without real-time panel-specific insights, there is a pressing need to advance these systems using robotic perception technologies. Current research endeavors aim to transition from rudimentary semi-automated processes to sophisticated robotic systems that can autonomously detect defects and anomalies on solar panels, thereby elevating the efficacy and reliability of inspection procedures.

The integration of unmanned aerial vehicles (UAVs) into industrial inspection frameworks represents a transformative approach, addressing inherent challenges and limitations of traditional inspection methods. The growing complexity and scale of industrial facilities necessitate advanced inspection techniques that can provide comprehensive, timely, and accurate assessments. UAV-based inspections offer distinct advantages, including enhanced accessibility to hard-to-reach areas, reduced operational costs, and minimized safety risks for personnel. Moreover, the deployment of UAVs enables real-time data acquisition and analysis, facilitating proactive maintenance and informed decision-making processes. By harnessing the capabilities of UAVs, industries can achieve a more streamlined, efficient, and adaptable inspection workflow, thereby optimizing asset management, ensuring regulatory compliance, and enhancing overall operational performance.

The visual perception system of a UAV is a critical component for this task, enabling the acquisition and analysis of the imagery data necessary for condition monitoring of solar modules. Traditional approaches to object detection in visual perception systems typically employ bounding box detection [4,5,6] or instance segmentation [7,8], or even classical approaches [9], to identify and delineate objects of interest within the imagery data. However, these methodologies can entail additional computational overheads when utilized for real-time processing on embedded platforms.

In this paper, we introduce REFIT (real-time farm inspection), a novel approach to object detection tailored for real-time solar farm inspections using UAVs. Our methodology hinges on the detection of keypoints, specifically the vertices of solar panels, which are structurally uniform and exhibit a rectangular shape. This approach facilitates efficient estimation of the six degrees of freedom (6DoF) pose of each panel using inexpensive methods such as Perspective-n-Point [10] (PnP), thereby providing crucial spatial information for the UAV’s navigation and operational planning. A distinct gap in the current literature is the prevalent focus on general object detection pipelines, which necessitate subsequent post-processing of the model output to obtain accurate spatial information. Addressing this limitation, our proposed framework provides an integrated solution that streamlines the detection process, minimizing the need for additional computational steps, while ensuring precise and timely spatial insights. Decisions might include adjusting the position to enhance image quality or utilizing these data to create a detailed map of a power plant. Such maps prove invaluable in the subsequent stages of industrial inspection, offering a comprehensive and accurate blueprint for further analysis and action.

By optimizing our architecture for an embedded platform, we are able to surpass the operational frequency of most cameras, ensuring real-time processing.

Furthermore, we have incorporated an uncertainty measure into our model, enabling the employment of active learning strategies to minimize the labeling efforts required during the training phase, addressing a common oversight in the realm of machine learning for visual perception systems.

The overarching goal of this endeavor was to devise a pragmatic and deployable model, without venturing into the realm of theoretical novelties, to provide a seamless and efficient inspection process for solar farms. Through our architecture, we aspire to strike a balance between computational efficiency and practical utility, ensuring the model serves as a robust onboard visual perception system for UAVs in real-world solar farm inspection scenarios.

This work centers around the development of a holistic solution for robotic perception in the context of industrial facility inspections, specifically solar farms. The salient contributions are:

1. **Keypoint-Based Detection System for Robotic Perception**: We introduce a novel object detection paradigm that identifies keypoints on solar panels, serving as an effective solution for real-time robotic perception during the inspection of industrial facilities.
2. **Efficient Onboard Architecture Tailored for Real-time UAV Processing**: We provide an efficient model specifically designed for real-time onboard computations on UAVs. This ensures that the UAV can instantly perceive and react to its surroundings, emphasizing the importance of responsiveness in industrial inspection scenarios.
3. **Uncertainty Metric with Active Learning Application**: To mitigate the labeling challenges that come with deploying deep learning models, we integrate an uncertainty metric into our system. This metric’s efficacy, when applied in an active learning context, was rigorously tested and validated, demonstrating its potential in reducing the labeled data required for effective model training.

The subsequent sections of this paper delve deeper into the nuances of our methodology and findings. In Section 2, we turn our attention to relevant studies, highlighting the dual avenues of robots employed for industrial inspections and contemporary real-time instance segmentation methodologies. Section 3 elucidates the design intricacies of our model and the genesis of the uncertainty metric. This is followed by Section 4, where we present a comprehensive analysis of our experiments, including comparative studies against benchmark methods and a dedicated subsection on our active learning experiment. The paper concludes with Section 5, offering a reflective discourse on our results and avenues for future work.

## 2. Related work

### 2.1. Autonomous Inspection of Industrial Facilities Using UAVs

Aerial robotics stands as a transformative force across various sectors, revolutionizing industrial inspection processes with its many advantages. UAVs have demonstrated exceptional proficiency in swiftly and accurately accessing challenging locations, while safeguarding industrial equipment’s integrity [11,12,13,14].

Despite ongoing efforts to automate industrial inspection, such as those explored in [15,16], these endeavors remain confined to laboratory settings. In practical inspection scenarios, remotely piloted aircraft systems (RPAS) are typically employed. Here, a pilot guides the UAV to capture pertinent data, commonly images or point clouds of objects of interest, which are subsequently processed to generate inspection reports [1]. Therefore, there is no online analysis of the data taken. It is after the inspection that the images collected are processed in order to detect defects or problems in the inspected structures.

In outdoor operations, like photovoltaic panel inspections, pilots predefine a series of GPS waypoints for the UAV [17]. Following these waypoints, the UAV captures images of the plant at specified intervals. While this approach streamlines the inspection process, it necessitates multiple passes over the same panels to ensure comprehensive coverage [14].

The ability to detect and extract panel positions while airborne holds significant potential. By doing so, the UAV’s flight path can be optimized, ensuring complete panel image acquisition during the inspection. Furthermore, having precise panel positions referenced in the images facilitates the subsequent inspection stages, leading to more informative and accurate inspection reports.

### 2.2. Real-Time Instance Segmentation

The pursuit of real-time instance segmentation has led to the emergence of a plethora of methodologies, each with unique architectural designs and operational mechanisms. Among these, YOLACT [18] (you only look at coefficients) and its successor YOLACT++ [19] stand out for their innovative approach of deconstructing the task into parallel subtasks: the generation of prototype masks, and the prediction of per-instance mask coefficients. These prototypes and coefficients were amalgamated to yield high-quality instance masks, with YOLACT++ further enhancing the processing speed and accuracy by incorporating deformable convolutions and optimizing the prediction head.

On a similar note, YolactEdge [20], a variant of YOLACT, introduced two pivotal enhancements. It employs TensorRT [21] optimization, to balance speed and accuracy, and unveiled a novel feature warping module that capitalizes on the temporal redundancy in videos to improve instance segmentation results. However, the module is tailored for video stream processing, contrasting with our objective of processing images on a one-to-one basis, ensuring accurate and independent solar panel detection in each frame.

The CenterPoly [22] architecture, along with its enhanced version, CenterPolyV2 [23], adopts a two-stage approach for real-time instance segmentation. In the first stage, objects are pinpointed using their center keypoints. Subsequently, a fixed number of polygon vertices are predicted for each detected object. CenterPolyV2 augments this strategy by incorporating a novel region-based loss and order loss. Additionally, it introduced an advanced training methodology for vertex prediction, showcasing substantial advancements on intricate datasets.

While there are evident parallels between our approach and CenterPoly, particularly in the utilization of fixed keypoints, the objectives and applications of the two methods diverge. Our model has been meticulously optimized for utmost speed and specifically designed for scenarios where a consistent number of keypoints are always discernible in the image. This tailored optimization ensures efficiency and precision in our targeted application of solar farm inspections using UAVs. In contrast, CenterPoly aims at broader applicability, focusing on the more generalized task of instance segmentation in diverse, uncontrolled environments.

These real-time instance segmentation methodologies, though not explicitly evaluated on embedded platforms, exhibit a rich tapestry of innovative approaches in processing single or consecutive image frames. Their design principles and operational mechanisms provide a comprehensive backdrop to our research endeavor focused on developing a robust real-time visual perception system for UAV-based solar farm inspections. Through a nuanced understanding of these methodologies, we aimed to bridge the apparent gap in evaluating real-time instance segmentation models on embedded platforms, which is essential for practical UAV operations in solar farm inspections.

### 2.3. Active Learning

Active learning [24], a paradigm of machine learning, seeks to optimize the model training process through judicious selection of unlabeled data for annotation. Instead of arbitrarily labeling all available unlabeled samples, active learning aspires to choose those data points that, once labeled, are expected to significantly enhance the model’s performance. This approach is particularly instrumental in scenarios where labeling data is expensive or time-consuming, thus necessitating a more efficient strategy for data annotation.

Within the active learning framework, every unlabeled instance *x* is attributed a metric v(x) to assess its prospective impact on enhancing the model’s performance. This metric can be inferred from the current model’s output and might also reflect the statistical attributes of the instance itself. A higher value of v(x) suggests a higher priority for selecting the instance, owing to its probable merit in honing the model, while a lower value might denote a lower selection priority.

One of the most common methodologies in active learning is uncertainty-based sampling. This approach exploits the uncertainty estimates from model predictions to pinpoint valuable samples for annotation. The underlying rationale is that regions where the model exudes uncertainty in its predictions are likely to be challenging or ambiguous cases. By annotating these uncertain instances, one can add additional information to the model, thereby elevating its performance, especially in complex tasks like object detection.

In the realm of deep object detection, uncertainty-based active learning metrics utilize various measures such as entropy or margin sampling predicated on class probability distributions per object proposal. These metrics quantify the confidence or certainty level of a model’s prediction for each sample and prioritize those exhibiting higher uncertainties for annotation.

A notable technique for estimating model uncertainty is Monte Carlo dropout [25]. This method entails sampling multiple predictions from a trained model with dropout enabled during inference. Dropout is applied to the weights of the neural network, and multiple forward passes through the network are executed, engendering different predictions for each pass. By averaging these predictions, an estimate of the model uncertainty is obtained. Employing Monte Carlo dropout for uncertainty estimation necessitates no modifications to the models.

## 3. Methods

### 3.1. Method Overview

CenterNet stands out in the domain of object detection architectures due to its ability to simplify traditionally complex pipelines into an efficient and straightforward paradigm. At its core, CenterNet employs a convolutional backbone and a series of task-dependent convolutional heads that cater to specific functionalities, such as center heat map localization, quantization error correction, and object dimension regression.

Our focus revolved around tailoring this architecture to the specialized needs of robotic perception. To that end, we performed several pivotal architectural modifications:

- **Backbone Alteration:** The backbone of a deep learning model plays a crucial role in determining its performance. Given the constraints associated with real-time processing on embedded platforms, we integrate a MobileNet-V3 backbone. This choice ensures computational efficiency, while retaining the capacity for robust feature extraction and representation.
- **Keypoint Regression:** Conventional bounding box regressors offer a generalized spatial perspective. However, robotic applications, with their emphasis on tasks like navigation, planning, and interaction, necessitate a more nuanced spatial understanding. By introducing a keypoint regressor head, we offer detailed spatial insights and pave the way for efficient 6DoF object pose estimation, employing methods such as Perspective-n-Points (PnP).
- **Pragmatic Deployment:** Deploying deep learning models in real-world scenarios often confronts the challenge of high data-labeling costs. In addressing this hurdle, our architecture incorporates an integrated uncertainty measure, laying the foundation for the incorporation of active learning strategies.

### 3.2. Problem Formulation

Consider an input image denoted by $I\in R^{W\times H\times 3}$. Our primary objective is to detect a collection S of structured objects. Each object o∈S can be uniquely identified using a set of *k* keypoints, $K_{o}$.

The foundational step involves constructing a center map ${\hat{Y}}_{\mathrm{center}}$ with dimensions ${\left[0,1\right]}^{W/R\times H/R\times C}$, where *C* symbolizes the number of distinct classes, and *R* is the output stride.

Given the intricacies of regressing a quantized center map directly, we apply a Gaussian filter with a standard deviation $\sigma_{c}$ to smooth out this map:

$$Y=G({\hat{Y}}_{\mathrm{center}},\sigma_{c}),$$

where G() signifies the Gaussian filtering procedure.

For center prediction, the focal loss is given by:

$$L_{\mathrm{center}}=-\frac{1}{n}\sum_{i}\left\{\begin{array}{cc} {\left(1-{\hat{y}}_{i}\right)}^{\alpha }\log\left({\hat{y}}_{i}\right) & \mathrm{if}\;y_{i}=1,\\ {\left(1-y_{i}\right)}^{\beta }{\left({\hat{y}}_{i}\right)}^{\alpha }\log\left(1-{\hat{y}}_{i}\right) & \mathrm{otherwise}. \end{array}\right.$$

To account for quantization errors originating from the output stride *R*, we introduce an offset map ${\hat{Y}}_{\mathrm{offset}}$. The values in this map, lying within [0,1], serve as fractional adjustments that rectify center locations. The corresponding loss for this regression is a piecewise-defined masked L1 loss:

$$L_{\mathrm{offset}}\left(x,y\right)=\left\{\begin{array}{cc} \left|Y_{\mathrm{offset}}\left(x,y\right)-{\hat{Y}}_{\mathrm{offset}}\left(x,y\right)\right| & \mathrm{if}\;Y_{\mathrm{center}}\left(x,y\right)=1,\\ 0 & \mathrm{otherwise}. \end{array}\right.$$

Similarly, the loss function for keypoint regression $L_{\mathrm{keypoints}}$ is defined as

$$L_{\mathrm{keypoints}}\left(x,y\right)=\left\{\begin{array}{cc} \left|Y_{\mathrm{keypoints}}\left(x,y\right)-{\hat{Y}}_{\mathrm{keypoints}}\left(x,y\right)\right| & \mathrm{if}\;Y_{\mathrm{center}}\left(x,y\right)=1,\\ 0 & \mathrm{otherwise}. \end{array}\right.$$

The overall loss function for our network is a weighted combination of the aforementioned components:

$$L=\lambda_{\mathrm{center}}L_{\mathrm{center}}+\lambda_{\mathrm{offset}}L_{\mathrm{offset}}+\lambda_{\mathrm{keypoints}}L_{\mathrm{keypoints}}$$

### 3.3. Network Architecture

To strike a balance between real-time performance and computational efficiency, our architectural foundation is rooted in a modified version of MobileNet-V3 [26] (specifically its small variant). Although MobileNet-V3 intrinsically reduces the input image resolution by a factor of 32, such substantial downsampling risks impairing spatial precision—particularly when pinpointing closely clustered or minuscule objects.

**Figure 1:**
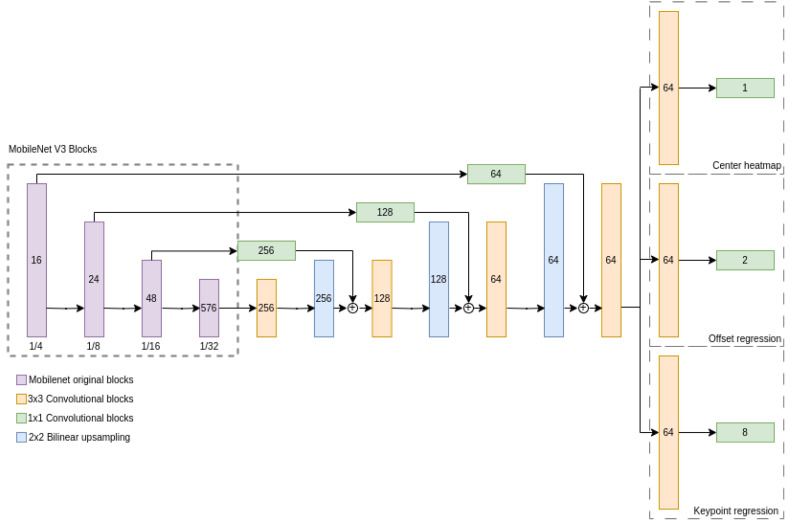
Proposed model architecture. MobileNetV3 small [26] forms the bedrock, facilitating efficient feature distillation. Due to the inherent stride of 32 in MobileNetV3’s output, a sequence of 3 × 3 convolutional segments and bilinear upsampling is introduced, culminating in a terminal stride of 4. The stature of each convolutional segment mirrors the contemporaneous resolution of the feature map, while the encased numeral signifies the kernel tally. Each segment is composed of a 2D convolution, succeeded by a hard sigmoid activation and a dropout mechanism.

To address this limitation, we incorporated several upsampling layers devised to amplify the feature resolution to a more congenial downsampling ratio of 4. This refinement procedure entails a trio of 3 × 3 convolutions, each succeeded by 2 × 2 bilinear upsampling. Recognizing the significance of preserving spatial integrity and amalgamating multi-scale features, we integrated residual skip connections. Drawing inspiration from the U-Net [27] framework, these connections are judiciously positioned after each bilinear upsampling phase. A 1 × 1 convolution adjusts the channel count of the initial feature map to align with the recipient layer’s channel dimension. In keeping with our commitment to swift processing, without undermining model competence, these skip connections were devised to be computationally frugal. Subsequent to the upsampling, we deploy a terminal 3 × 3 convolution to counteract the emergence of potential artifacts. The complete architecture is illustrated in Figure 1.

Following the primary feature map extraction by the backbone, we incorporate bespoke task heads. A typical head encompasses an inaugural 3 × 3 convolution comprising 64 filters, succeeded by a 1 × 1 convolution, designed to cater to the specific output channel requirements of each head. The hard sigmoid function has been adopted as the predominant activation strategy across the architecture. Moreover, succeeding each convolution is a batch normalization layer, poised to harmonize activations and enhance training kinetics. To augment the model resilience, dropout layers (with a retention probability of 0.5) are interspersed after each convolution. This not only bestows regularization but paves the way for potential Monte Carlo dropout techniques for uncertainty quantification.

Every architectural decision resonates with our endeavor to curtail the computational demand while preserving the model’s operational suitability for instantaneous solar farm inspections using UAVs.

### 3.4. Uncertainty Estimation and Active Learning

Uncertainty estimation is an essential component in active learning, especially when aiming for continual model improvement with limited labeled data. For UAV-based solar farm inspections, gauging the model’s confidence in its predictions becomes vital. Monte Carlo dropout leverages dropout layers in a neural network during inference, not just during training. By conducting multiple forward passes with dropout activated (even in evaluation mode), an ensemble of models is effectively created. Given an input image, our model runs in evaluation mode with dropout activated 100 times, simulating an ensemble of 100 models.

For each of these 100 Monte Carlo iterations, a heat map output, denoted as *H*, is derived. To counter the model’s tendency towards low contrast outputs for challenging images, we employ a percentile-based normalization approach:

$$H_{\mathrm{normalized}}=\frac{H-\mathrm{percentile}(H,p_{1})}{\mathrm{percentile}\left(H,p_{2}\right)-\mathrm{percentile}\left(H,p_{1}\right)}$$

where $p_{1}$ and $p_{2}$ are the lower and upper percentiles, respectively. Following this, the standard deviation, denoted as σ, for each pixel across all passes is calculated. A larger σ indicates a variance among the passes for that specific pixel. Our heat map uncertainty measure, $U_{\mathrm{heatmap}}$, is determined using the 95th percentile of these standard deviations.

For offsets and keypoints, the approach is slightly modified. For each respective output, the standard deviation for every channel across the 100 passes is computed. The maximum standard deviation from all channels is selected. The 95th percentile of these maximum standard deviations gives the uncertainty measure for that particular output.

Integrating the individual uncertainty measures for the heat map, offsets, and keypoints, the cumulative uncertainty is computed as

$$U=\lambda_{\mathrm{heatmap}}\times U_{\mathrm{heatmap}}+\lambda_{\mathrm{offsets}}\times U_{\mathrm{offsets}}+\lambda_{\mathrm{keypoints}}\times U_{\mathrm{keypoints}}$$

where the λ values are the weights used during training to balance the loss terms.

**Figure 2:**
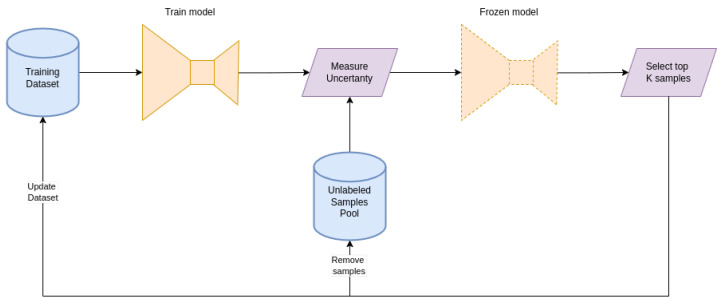
Active learning workflow. The process starts with the training of a model using a minimal labeled dataset. Post training, the model evaluates the complete pool of unlabeled samples, estimating the uncertainty. The top *k* samples exhibiting the highest uncertainty are selected for labeling and subsequently integrated into the training dataset. This iterative cycle continues until the optimal performance or a predefined criterion is met.

This uncertainty estimation approach aligns impeccably with the active learning framework. By identifying high uncertainty data points, they are prioritized for labeling, refining the model’s learning from a limited labeled dataset. The efficacy of this strategy, especially in reducing the demand for labeled data, will be expanded upon in the experimental results section. Although our method is not crafted for real-time uncertainty estimation, it provides a formidable mechanism for active learning in UAV-based solar farm inspections. Figure 2 depicts a diagram of the proposed pipeline.

## 4. Results

### 4.1. Dataset

**Figure 3:**
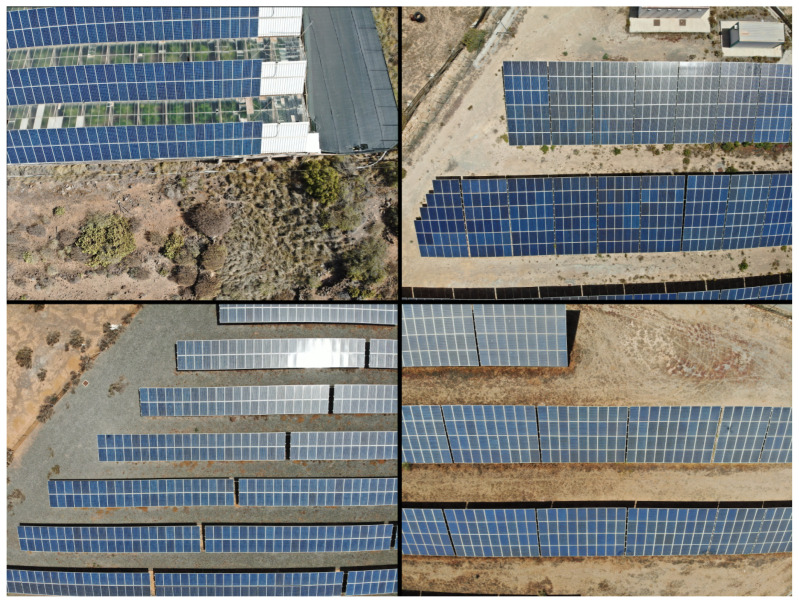
Exemplars from our test dataset, elucidating the dense arrangement and diminutive size of the objects. The multitude of objects, numbering in the hundreds, poses a non-trivial challenge for real-time detection.

A comprehensive dataset was meticulously collected from real inspection flights conducted by a designated company, adhering strictly to prevailing regulations. These aerial inspections were performed at an elevation of 40 m employing a DJI Matrice 300 equipped with a DJI Zenmuse H20T. The dataset covers over 240,000 solar panels, illustrating a substantial breadth of data. All images were systematically resized to dimensions 1024 × 1376 pixels, ensuring an adequate resolution to distinctly identify all solar panels within the images. Some samples of the dataset are shown in the Figure 3.

### 4.2. Implementation Details

Our models were trained on a workstation with two NVIDIA RTX 2080 Ti GPUs. The implementations were carried out utilizing the PyTorch [28] and PyTorch Lightning [29] frameworks. The training regimen employed the AdamW [30] optimizer, initialized with a learning rate of $10^{-3}$. A cosine annealing learning rate scheduler was utilized, alongside a weight decay parameter of 0.01. The data augmentation pipeline was judiciously simplistic, encompassing contrast adjustments, minor hue shifts, and brightness modifications, eschewing spatial transformations like flips, shears, and zooms to prevent object deformation.

### 4.3. Comparative Analysis

**Figure 4:**
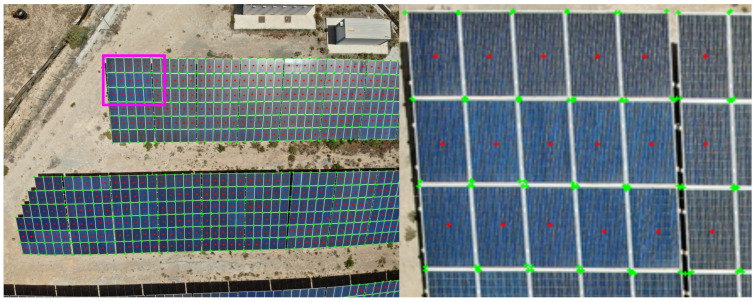
Detection results. On the (**left**), full image of 1024 × 1376 provided to the detector. On the (**right**), a zoom in of the magenta bounding box. Object center detections are depicted with red dots, while the green dots depict the detected keypoints per object.

The task that most aligns with our objective in the domain of deep learning models is instance segmentation. We engaged in a rigorous comparative analysis against contemporary real-time instance segmentation models, namely CenterPoly, CenterPolyv2, YOLACT, and YOLACT++. The code provided for each model was utilized verbatim, adhering to all specified prerequisites. We did not perform any hyperparameter tuning, retaining them as per the default specifications outlined in the respective public repositories, with the exception of minimal alterations for training on our dataset. For CenterPoly and its variant, the lightweight Hourglass-104 architecture was employed to reduce computational overheads, whereas for YOLACT and YOLACT++, two variants were trained; one with a Resnet-101 backbone and another with Resnet-50 to optimize speed. All models were designed for real-time instance segmentation on desktop GPUs. Given our objective of real-time detection on UAV-embedded platforms, the NVIDIA AGX Jetson Orin platform was chosen as the test bench. This potent computing platform is compatible with larger drones like the DJI Matrice 300. Table 1 encapsulates the results. Our model, devoid of the need for extensive post-processing like non-maximum suppression, only required the network inference time measurement, thereby avoiding an unfavorable portrayal of competing models. Even under these lenient latency measurements, our model outperformed all other models by a substantial margin, being 3.45-times faster than the next fastest compared method and achieving a throughput twice that of the common camera frame rate of 30FPS. Moreover, our model exhibited superior performance for average precision (AP) metrics. By optimizing our architecture for an embedded platform, specifically NVIDIA Jetson AGX Orin, we achieved a processing rate of almost 60 frames per second (FPS) at a resolution of 1024 x 1376 pixels, thereby surpassing the operational frequency of most cameras and ensuring real-time processing. Figure 4 depicts the output of the model of a given sample.

### 4.4. Uncertainty-Based Active Learning

Active learning, by design, leverages existing labeled data to train a model, which is then deployed over the entire pool of unlabeled data. The primary objective is to compute a metric that gauges the utility of each unlabeled sample in terms of its potential contribution once labeled. After determining these high-potential samples, they are labeled and incorporated into the training dataset, and the model is subsequently retrained. This iterative process is sustained until a predefined stopping criterion is met, which might include reaching a specific metric threshold on the test set or the labeling of a maximum number of samples.

One of the paramount challenges in active learning is the derivation of a metric that can pinpoint the samples where the model commits the most errors. This ideal metric would require access to true labels, making it infeasible in real-world settings. Given this backdrop, we hypothesized that our constructed uncertainty metric, despite its simplicity, could serve as an effective proxy for the actual error. In essence, we postulate that samples manifesting higher uncertainty are likely those where the model errs the most.

To validate this hypothesis, a series of experiments were conducted. Our initial dataset comprised a randomly selected 5% of the available data. Upon training, the final loss was computed on the test dataset. Thereafter, in increments of 20% of the dataset’s size, new samples were incorporated into the training subset, followed by model retraining. The sample selection process was guided by three distinct strategies: (i) selecting samples with the most pronounced loss (not feasible in realistic scenarios due to the prerequisite of labels to compute the loss), (ii) opting for samples with the highest uncertainty, and (iii) random sample selection. To enhance the robustness of our findings, this procedure was replicated ten times, and the results were averaged to mitigate the influence of outliers and anomalies.

**Figure 5:**
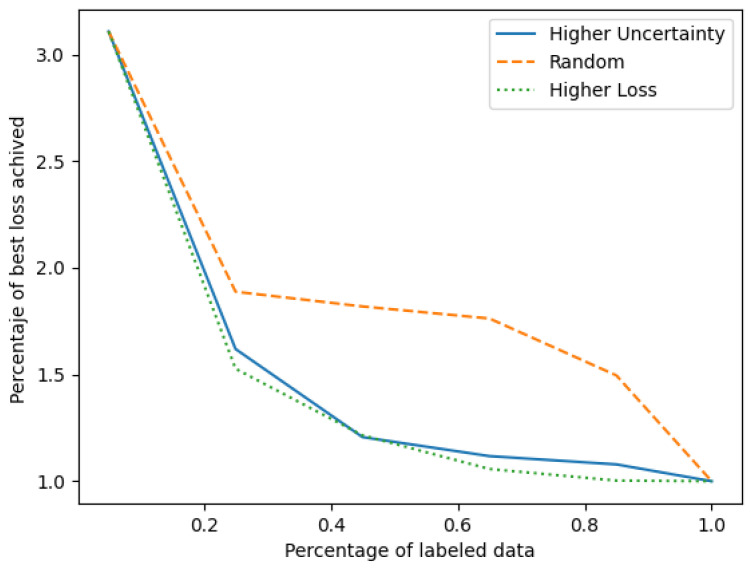
Evolution of the test set loss relative to the model trained on the entire dataset, as a function of the percentage of labeled data. The comparison among three sample selection strategies: higher uncertainty, higher loss, and random selection, showcases the efficacy of uncertainty-based selection in achieving a comparable performance with a reduced labeled dataset.

Figure 5 summarizes our findings. The horizontal axis delineates the percentage of labeled data used, while the vertical axis portrays the relative loss of the test set relative to the loss procured using the entirety of the dataset.

Upon analyzing the figure, it is apparent that the uncertainty-based selection strategy closely parallels the results of the loss selection, which is unattainable in real-world scenarios, due to the unavailability of true labels. Remarkably, our uncertainty-driven approach achieved competitive performance using only 25% of the dataset. On the contrary, a random sampling strategy only reached a similar efficiency when using around 85% of the data. This underscores the capability of our uncertainty metric to discern and prioritize information-rich samples amid extensive and potentially redundant data, presenting substantial labeling cost reductions in real-world, large-scale dataset scenarios.

In summation, our active learning experiments underscored the practicality and potency of an uncertainty-based data selection strategy for our model, corroborating our initial hypothesis.

## 5. Discussion

Our research into keypoint-based object detection presents a compelling approach for real-time perception in robotic solar farm inspection missions. Rather than using traditional bounding box- or segmentation-based methods, our methodology is based on the detection of the vertices of solar panels. This distinction is critical, especially in the context of pose estimation, which is indispensable for precise UAV navigation and planning.

Compared with the leading real-time instance segmentation models, the advantages of our method are evident (Table 1). REFIT surpassed its contemporaries in both processing speed and accuracy metrics. Such a performance is particularly commendable when realized on UAV-embedded platforms like the NVIDIA AGX Jetson Orin.

We employed these established methods with minimal modifications to align them with our dataset. These models, designed for general-purpose instance segmentation and capable of handling multiple overlapping objects, were not optimized for our specific task. In contrast, our model was meticulously crafted for solar panel detection, striking a balance between computational complexity (and thus inference speed) and accuracy. This careful design resulted in a model perfectly tailored for our task, minimizing the susceptibility to overfitting. Despite utilizing the smallest available backbone, the original models, being designed for more general applications, were larger and more prone to overfitting on our specific dataset.

Additionally, our approach capitalizes on the inherent four-sided polygonal nature of solar panels, making our representation more efficient than a traditional mask. This efficiency minimizes inaccuracies and the need for the model to output redundant information, thereby simplifying the training process. Notably, fine-tuning hyperparameters for the alternative models falls outside the scope of our work. However, neglecting this optimization widens the performance gap between these models and ours. Although hyperparameter tuning could potentially narrow this gap, our model maintains a clear advantage for this specific solar panel detection problem.

Moreover, our model facilitates asset pose estimation using conventional methods, a task that proves challenging and potentially less robust with the other alternatives. While the alternative approaches might require post-processing the mask to extract keypoints, our model inherently supports this pose estimation task, even in scenarios involving shapes other than four-sided polygons or non-planar objects. While untested in such scenarios, our model’s design accounts for their potential complexity, and we anticipate its adaptability.

Conversely, our model is not tailored for general-purpose instance segmentation. Presently, it can only detect objects when all corners of the solar panels are visible in the image. While this limitation is critical for general-purpose instance segmentation, it is inconsequential for industrial facility inspections where the robot adheres to a predetermined plan, ensuring complete visibility of solar panels during the inspection process.

Furthermore, the architectural decisions of REFIT are grounded in pragmatism. By prioritizing reduced computational demands, our model emerges as a viable choice for real-world applications. Our design is a demonstration of our dedication to effective learning, incorporating active learning principles, promising significant reductions in annotation efforts, and bolstering the model’s applicability in practical settings.

## 6. Conclusions

In conclusion, our research pioneers a revolutionary approach to real-time perception in robotic solar farm inspection missions by adopting keypoint-based object detection. Departing from traditional bounding box- or segmentation-based methods, our innovative strategy focuses on detecting the vertices of solar panels, addressing the crucial need for precise pose estimation and thereby enhancing UAV navigation and planning accuracy.

A notable achievement is the development of REFIT, a model surpassing the leading real-time instance segmentation models in both processing speed and accuracy metrics, as outlined in Table 1. REFIT’s exceptional performance is accentuated when deployed on UAV-embedded platforms, such as the NVIDIA AGX Jetson Orin, highlighting its practical applicability in real-world scenarios.

Moreover, REFIT’s architectural foundations prioritize pragmatism and computational efficiency. Carefully managing computational requirements, while optimizing for performance metrics, well positions our model for effective real-world applications, bridging theoretical advancements with practical implementation.

Looking forward, our research can act as a catalyst for future exploration in UAV-based solar farm inspections. We foresee promising avenues, including region-specific active learning, field trials for authenticating model robustness, and the integration of direct pose estimation to enhance inspection insights.

In essence, our research signifies a paradigm shift in UAV-based solar farm inspections, seamlessly combining computational efficiency with stringent performance benchmarks. By elucidating potential trajectories for future research, we lay the groundwork for ongoing advancements in this field. As a testament to our commitment to academic rigor and collaborative progress, our codebase is publicly accessible at https://github.com/cvar-upm/REFIT (accessed on 24 January 2024), fostering collaboration, reproducibility, and sustained progress in this vital domain.

While comprehensive, our work serves as a beacon for future exploration, highlighting the following potential trajectories:

- Region-specific active learning: Exploring active learning based on image-specific regions holds promise. Addressing uncertainties in occluded areas, contrasted with clearer regions, could refine the model efficiency and accuracy, further reducing labeling costs.
- Field trials: Authenticating our model’s robustness through real-world UAV inspection flights is paramount, providing insights and highlighting potential areas for improvement.
- Direct pose estimation: Enriching our model with integrated pose estimation could increase inspection depth, offering enhanced navigational insights for UAVs.

## Figures and Tables

**Table 1:** Comparative results sequenced in descending FPS. All models were trained employing their respective code releases and specifications on the Nvidia Jetson AGX Orin.

Method	Backbone	FPS	Runtime	AP	AP50	AP75
Centerpoly	Hourglass-104	1.9	540	36.7	80.4	76.5
CenterpolyV2	Hourglass-104	1.9	540	35.4	80.7	73.25
YOLACT++	Resnet-101-DCNv2+FPN	11.2	89	57.5	89.1	57.7
YOLACT	Resnet-101+FPN	13.4	72	47.0	80.3	45.3
YOLACT++	Resnet-50-DCNv2+FPN	14.1	71	55.2	88.0	56.8
YOLACT	Resnet-50+FPN	17.0	59	45.3	77.6	44.0
REFIT (Ours)	MobileNetV3 small	**58.8**	**17**	**74.7**	**96.5**	**88.5**

## Data Availability

Due to industrial confidentiality and industrial property, the data supporting the findings of this study cannot be made publicly available. The dataset for this research was provided by DataDron and is subject to confidentiality agreements. Limitations on data sharing are necessitated by the constraints of the consent provided by the participating entity.

## Abbreviations

The following abbreviations are used in this manuscript:

AP	Average Precision
FPS	Frames Per Second
DoF	Degrees of Freedom
GPU	Graphical Processing Unit
PnP	Perspective-n-Point
REFIT	Real-Time Farm Inspection
RPAS	Remotely Piloted Aircraft System
UAV	Unmanned Aerial Vehicle
